# Anti-gout Potential of Malaysian Medicinal Plants

**DOI:** 10.3389/fphar.2018.00261

**Published:** 2018-03-23

**Authors:** Fazleen I. Abu Bakar, Mohd F. Abu Bakar, Asmah Rahmat, Norazlin Abdullah, Siti F. Sabran, Susi Endrini

**Affiliations:** ^1^Faculty of Applied Sciences and Technology, Universiti Tun Hussein Onn Malaysia, Muar, Malaysia; ^2^Centre of Research for Sustainable Uses of Natural Resources, Universiti Tun Hussein Onn Malaysia, Parit Raja, Malaysia; ^3^Faculty of Medicine, YARSI University, Jakarta, Indonesia

**Keywords:** xanthine oxidase inhibition, anti-gout, phytochemical, Malaysian medicinal plants, *in vitro*, *in vivo*

## Abstract

Gout is a type of arthritis that causes painful inflammation in one or more joints. In gout, elevation of uric acid in the blood triggers the formation of crystals, causing joint pain. Malaysia is a mega-biodiversity country that is rich in medicinal plants species. Therefore, its flora might offer promising therapies for gout. This article aims to systematically review the anti-gout potential of Malaysian medicinal plants. Articles on gout published from 2000 to 2017 were identified using PubMed, Scopus, ScienceDirect, and Google Scholar with the following keyword search terms: “gout,” “medicinal plants,” “Malaysia,” “epidemiology,” “*in vitro,”* and “*in vivo*.” In this study, 85 plants were identified as possessing anti-gout activity. These plants had higher percentages of xanthine oxidase inhibitory activity (>85%); specifically, the *Momordica charantia, Chrysanthemum indicum, Cinnamomum cassia, Kaempferia galanga, Artemisia vulgaris*, and *Morinda elliptica* had the highest values, due to their diverse natural bioactive compounds, which include flavonoids, phenolics, tannin, coumarins, luteolin, and apigenin. This review summarizes the anti-gout potential of Malaysian medicinal plants but the mechanisms, active compounds, pharmacokinetics, bioavailability, and safety of the plants still remain to be elucidated.

## Background

**Figure 1:**
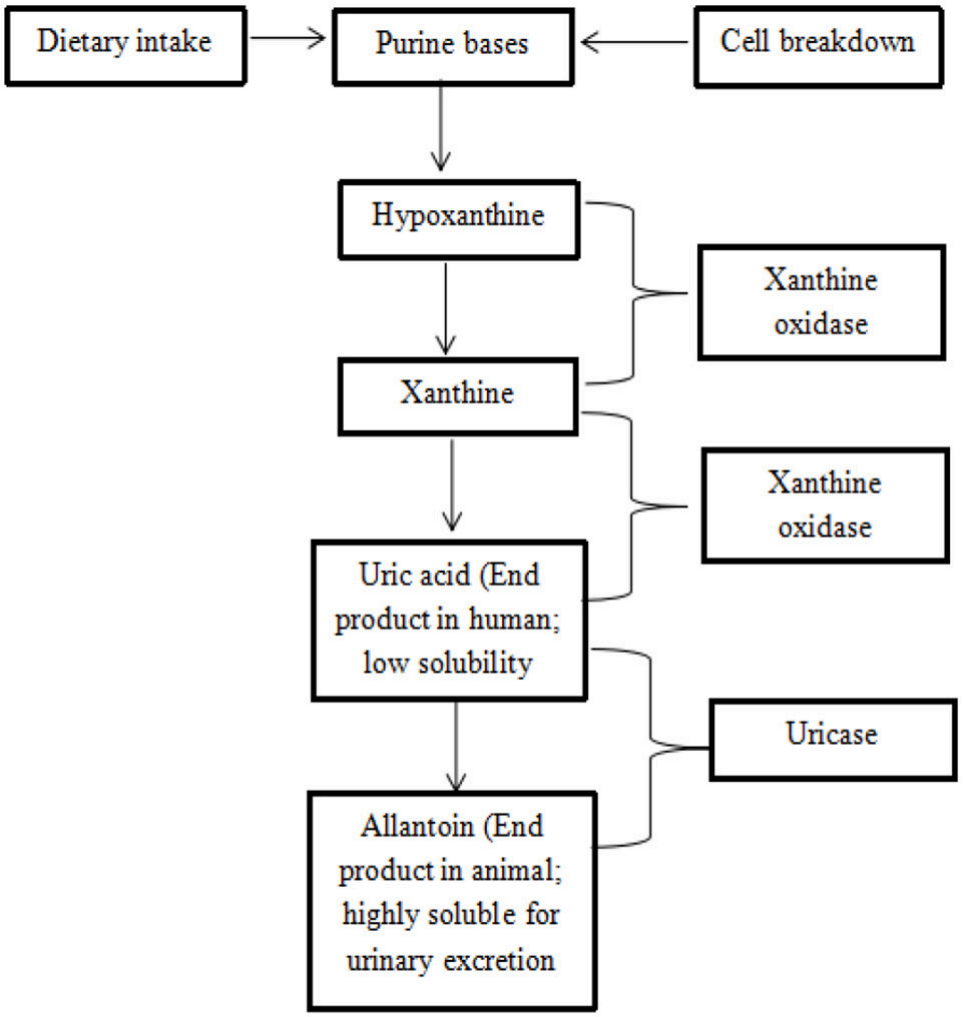
Mechanism of purine catabolism (Offermanns and Rosenthal, 2008; Bustanji et al., 2011).

Gout incidence has increased over the past 50 years, especially in developing countries (Kuo et al., 2015). Gout is a type of inflammatory arthritis triggered by interactions between monosodium urate (MSU) crystals and tissue (Dalbeth et al., 2014) during purine catabolism by the enzyme of xanthine oxidase (Nile et al., 2013). Xanthine oxidase catalyzes the oxidative hydroxylation of hypoxanthine to xanthine to uric acid, leading to painful inflammation (Nile and Khobragade, 2011). Uricase is an enzyme that further catalyzes the conversion of uric acid to the highly soluble allantoin that is excreted in the urine (Figure 1). Unfortunately, uricase is not a functional human enzyme and, as a result, humans can develop hyperuricemia (Gliozzi et al., 2016). Gout has also been reported to cause tophi, joint deformities, and kidney stones (Teh et al., 2014).

Hyperuricemia, a major etiological factor of gout, develops either due to overproduction caused by a metabolic disorder or due to under excretion of blood uric acid due to abnormal renal urate transport activity (Ichida et al., 2012). Kidney is the main regulator of serum uric acid levels where renal urate excretion is determined by the balance of the reabsorption and secretion of urate. Renal urate reabsorption is mainly mediated by two urate transporters—urate transporter 1 (URAT1) and glucose transporter 9 (GLUT9) (Enomoto et al., 2002; Matsuo et al., 2008). One of the mechanisms involved in reducing the plasma uric acid concentration is an inhibition of the reabsorption of urate in renal tissue via renal mRNA and protein levels of urate transporter 1 (URAT1), glucose transporter 9 (GLUT9), organic anion transporter 1 (OAT1) and organic cation/carnitine transporters (OCT1/2, OCTN1/2) (Sungthong et al., 2016). Hyperuricemia occurs when serum uric acid levels are >0.42 mmol/L (Stamp et al., 2007). Therefore, reducing uric acid is the main approach for the treatment of gout, with target levels of serum uric acid of less than 0.36 mmol/L (Falasca, 2006; Pillinger et al., 2007).

Several risk factors for the development of gout have been established, including hyperuricemia, age, genetic factors, dietary factors, alcohol consumption, metabolic syndrome, hypertension, obesity, diuretic use, cholesterol level, and chronic renal disease (Roddy and Doherty, 2010). Men are believed to have four- to nine-fold increased the risk of developing gout compared to women; however, once women reach menopause, they tend to develop gout, as the uricosuric action of estrogen is lost (Tausche et al., 2009). Genetics and race may also be important factors that contribute to the incidence of gout (Mohd et al., 2011).

Several drugs are approved for the treatment of gout, including colchicine, steroids, non-steroidal anti-inflammatory drugs (ibuprofen, naproxen, indomethacin, and aspirin), cyclooxygenase 2 (COX-2) inhibitors (etoricoxib), and allopurinol. Although these agents are effective, they also cause side effects, such as skin allergies, fever, rash, renal dysfunction, aseptic meningitis, and hepatic dysfunction (Nguyen et al., 2004; Strazzullo and Puig, 2007). For example, allopurinol, which is the most commonly used xanthine oxidase inhibitor for gout (Pacher et al., 2006), causes nephrolithiasis, hypersensitivity reaction, Stevens-Johnson syndrome, renal toxicity, allergic reactions, and fatal liver necrosis, and increases the toxicity of 6-mercaptopurin (Kong et al., 2004; Wang et al., 2004).

Recently, treating disease using medicinal plants is gaining new interest (Unno et al., 2004) and research on medicinal plants has increased worldwide (Tapsell et al., 2006; Triggiani et al., 2006) due to fewer side effects and lower costs (Srivastava et al., 2012). Malaysia is a country that has more than 8,000 species of flowering plants and ~7,411 plant species have been identified in Sabah, Malaysia Borneo; in addition, 1,300 medicinal plant species have been documented in Peninsular, Malaysia (Kulip, 2003; Abd Aziz et al., 2011). The aim of the present review is to provide comprehensive information on the potential of anti-gout Malaysian medicinal plants and review the scientific data, including the experimental methodologies, active compounds, and mechanisms of action against gout.

## Methods

PubMed, Scopus, ScienceDirect and Google Scholar databases were searched for publications from 2000 to 2017 with *in vitro* and *in vivo* data on Malaysian medicinal plants for gout. The search terms included the following: “gout,” “medicinal plants,” “*in vivo*,” “*in vitro*,” “epidemiology,” “Malaysia,” and “mechanisms.” Publications with available abstracts were also reviewed and ~99 publications, including journal articles and proceedings, were reviewed. Data from these studies were then were summarized (Table 1: *in vitro* data; Table 2: *in vivo* data).

**Table 1:** The medicinal plants which are considered to possess anti-gout activity based on *in vitro* studies.

**Scientific name**	**Family**	**Local name**	**Part/Solvent used**	**IC_50_ (μg/ml)**	**Xanthine oxidase inhibition**	**Active compounds**	**Reference(s)**
*Acorus calamus*	Araceae	Pokok jerangau	Rhizome/Methanol	89.2	55.10% at 100 μg/ml	NA	Nguyen et al., 2004
*Adenanthera payonina*	Leguminosae	Saga	Leaves/Methanol	NA	47.15% at 100 μg/ml	Cardiac glycosides	Apaya and Chichioco-Hern, 2011
*Allium ampeloprasum*	Liliaceae	Bawang perai	Leaves/Ethanol	NA	43.71% at 100 μg/ml	NA	El-Rahman and Abd–Elhak, 2015
*Alpinia galanga*	Zingiberaceae	Lengkuas	Rhizome/Ethanol	NA	57.99% at 100 μg/ml	NA	Yumita et al., 2013
*Annona muricata*	Annonaceae	Durian belanda	Leaves/Ethanol	>200	14.18% at 100 μg/ml	NA	Sunarni et al., 2015
*Annona reticulata*	Annonaceae	Lonang, Nona kapri	Leaves/Ethanol	171.73	47.38% at 100 μg/ml	NA	Sunarni et al., 2015
*Annona squamosa*	Annonaceae	Buah nona	Leaves/Ethanol	>200	6.37% at 100 μg/ml	NA	Sunarni et al., 2015
*Apium graveolens*	Apiaceae	Saderi	Leaves/Ethanol	NA	73.89% at 100 μg/ml	NA	El-Rahman and Abd–Elhak, 2015
			Leaves/Methanol	NA	37.92% at 100 μg/ml		Alsultanee et al., 2014
*Artemisia vulgaris*	Asteraceae	Baru cina	Leaves/Methanol	14.7	89.30% at 100 μg/ml	Flavonoids	Nguyen et al., 2004
*Averrhoa carambola*	Oxalidaceae	Belimbing manis	Leaves /Ethanol	NA	23.61% at 100 μg/ml	NA	Azmi et al., 2012
			Flowers/Ethanol		2.47% at 100 μg/ml		
			Ripe fruit peels/ethanol		7.11% at 100 μg/ml		
*Barleria prionitis*	Acanthaceae	Bunga landak	Folium/Ethanol	NA	1.73% at 100 μg/ml	NA	Yumita et al., 2013
*Barringtonia racemosa*	Lecythidaceae	Putat	Leaves/Methanol	NA	58.82% at 1,000 μg/ml	NA	Osman et al., 2016
			Endosperm/Methanol		57.20% at 1,000 μg/ml		
			Pericarp/Methanol		57.99% at 1,000 μg/ml		
			Infloresence axis/Methanol		59.54% at 1,000 μg/ml		
*Blumea balsamifera*	Asteraceae	Pokok Sembung, capa, telinga kerbau	Leaves/Methanol	0.111	NA	Flavonoids	Nessa et al., 2010
				6.0	80.90% at 100 μg/ml	NA	Nguyen et al., 2004
*Brassica oleracea*	Brassicaceae	Kubis merah	Leaves/Water	230,150.00	53.72% at 250 mg/ml	Phenolic acids, anthocyanins	Al-Azzawie and Abd, 2015
*Butea monosperma*	Fabaceae	Palasa	Roots/Methanol	5.0	75.00% at 100 μg/ml	NA	Nile and Park, 2014
*Caesalpinia sappan*	Caesalpiniaceae	Sepang	Wood/Methanol	14.2	78.50% at 100 μg/ml	NA	Nguyen et al., 2004
*Calophyllum inophyllum*	Calophyllaceae	Penaga laut	Leaves/Methanol	NA	25.63% at 100 μg/ml	Phenolic, tannins, flavonoids	Apaya and Chichioco-Hern, 2011
*Cantella asiatica*	Umbelliferae	Pegaga	Whole plant/Methanol	NA	27.20% at 100 μg/ml	NA	Nguyen et al., 2004
					41.00% at 100 μg/ml	NA	Kong et al., 2000
*Carica papaya*	Caricaceae	Betik	Leaves/Ethanol	NA	78.38% at 100 μg/ml	NA	Azmi et al., 2012
			Petioles/Ethanol		8.11% at 100 μg/ml		
			Seeds/Ethanol		19.82% at 100 μg/ml		
			Unripe fruits/Ethanol		68.47% at 100 μg/ml		
			Flowers/Ethanol		66.03% at 100 μg/ml		
			Unripe fruit peels/ethanol		71.17% at 100 μg/ml		
*Cassia fistula*	Fabaceae	kayu raja	Leaves/Methanol	NA	61.90 % at 100 μg/ml	Alkaloid, tannins	Apaya and Chichioco-Hern, 2011
			Seeds/Methanol		64.56% at 100 μg/ml		Jothy et al., 2011
*Chrysanthemum indicum*	Asteraceae	Bunga kekwa	Flower/Methanol	22	95.00% at 100 μg/ml	Luteolin and apigenin	Kong et al., 2000
*Chrysanthemum sinense*	Asteraceae	Teh bunga	Flower/Methanol	5.1	82.90% at 100 μg/ml	Caffeic acid, luteolin, eriodictyol	Nguyen et al., 2004
*Cinnamomum cassia*	Lauraceae	Kayu manis cina	Twig/Methanol	18	93.00% at 100 μg/ml	Eugenol	Kong et al., 2000; Nguyen et al., 2004
			Bark/Methanol	58	89.00% at 100 μg/ml		
				82.4	55.80% at 100 μg/ml		
*Cinnamomum cinnamon*	Lauraceae	Kayu manis	Leaves/Methanol	NA	44.34% at 100 μg/ml	NA	Alsultanee et al., 2014
*Citrullus colocynthis*	Cucurbitaceae	Tembikai	Seeds/water	NA	14.40% at 200 μg/ml	NA	Bustanji et al., 2011
*Citrus sinensis*	Rutaceae	Oren	Fruit shell/Methanol	NA	51.00% at 100 μg/ml	NA	Kong et al., 2000
*Clinacanthus nutans*	Acanthaceae	Belalai gajah	Aerial part/Ethanol	10	NA	NA	Tu et al., 2014
*Cucurbita pepo*	Cucurbitaceae	Labu	Seeds/methanol	NA	27.33% at 100 μg/ml	NA	Alsultanee et al., 2014
*Curcuma longa*	Zingiberaceae	Kunyit	Whole plant/methanol	NA	28.31% at 100 μg/ml	NA	Alsultanee et al., 2014
*Cymbopogon citratus*	Poaceae	Serai makan	Stalks/Eessential oil	NA	81.34% at ratio of volume concentration of essential oil per volume of solvent, 1:2	NA	Mirghani et al., 2012
*Cymbopogon nardus*	Poaceae	Serai wangi	Petiolum/Ethanol	NA	18.12% at 100 μg/ml	NA	Yumita et al., 2013
*Cyperus rotundus*	Cyperaceae	Rumput halia hitam	Rhizome/Methanol	52.9	79.40% at 100 μg/ml	NA	Nguyen et al., 2004
*Dimocarpus longan*	Sapindaceae	Longan	Flower/Ethyl acetate	115.8	78.60% at 100 μg/ml	Proanthocyanidin A2, Acetonylgeraniin A	Sheu et al., 2016
			Pericarps/Ethyl acetate	118.9	79.20% at 50 μg/ml		
			Twigs/Ethyl acetate	125.3	79.20% at 50 μg/ml		
			Seeds/Ethyl acetate	262.5	78.90% at 50 μg/ml		
			Leaves/Ethyl acetate	331.1	42.10% at 100 μg/ml		
*Dimocarpus longan malesianus*	Sapindaceae	Mata kucing, Longan hijau Sarawak	Leaves/Ethanol	NA	46.88% at 100 μg/ml	NA	Azmi et al., 2012
			Ripe fruit peels/Ethanol		13.41% at 100 μg/ml		
*Erythrina indica*	Fabaceae	Dedap batik	Bark/Methanol	52.75	NA	Phenolic	Sowndhararajan et al., 2012
*Erythrina stricta*	Fabaceae	Bunga dedap	Leaves/Chloroform fraction	21.20	NA	Phenolic and flavonoid	Umamaheswari et al., 2009
			Leaves/Ethyl acetate fraction	44.90			
*Glycyrrhiza uralensis*	Fabaceae	Akar manis	Root/Methanol	54.9	64.40% at 100 μg/ml	NA	Nguyen et al., 2004
*Hedyotis diffusa*	Rubiaceae	Rumput lidah ular	Aerial part/Methanol	78.9	55.90% at 100 μg/ml	NA	Nguyen et al., 2004
*Hibiscus sabdariffa*	Malvaceae	Asam susur	Calyx/Water	NA	19.40% at 200 μg/ml	NA	Bustanji et al., 2011
			Calyx/Ethanol	NA	27.12% at 200 μg/ml	NA	Wahyuningsih et al., 2016b
*Justicia gendarussa*	Acanthaceae	Daun rusa	Folium/Ethanol	NA	18.48% at 100 μg/ml	NA	Yumita et al., 2013
*Kaempferia galangal*	Zingiberaceae	Cekur	Rhizome/Ethanol	NA	28.86% at 100 μg/ml	NA	Yumita et al., 2013
			Rhizome/Methanol	53.4	90.60% at 100 μg/ml	NA	Nguyen et al., 2004
*Kalanchoe pinnata*	Crassulaceae	Setawar	Aerial part/Methanol	40.8	68.10% at 100 μg/ml	NA	Nguyen et al., 2004
*Lantana camara*	Verbenaceae	Bunga tahi ayam	Folium/Ethanol	NA	17.17% at 100 μg/ml	NA	Yumita et al., 2013
*Manilkara zapota*	Sapotaceae	Duku	Leaves/Ethanol	NA	70.81% at 100 μg/ml	NA	Azmi et al., 2012
			Peels/Ethanol		41.03% at 100 μg/ml		
			Seeds/Ethanol		11.81% at 100 μg/ml		
*Melaleuca leucadendra*	Myrtaceae	Gelam, kayu putih	Stem and fruit/Methanol	76.7	64.60% at 100 μg/ml	NA	Nguyen et al., 2004
*Mimosa pudica*	Leguminosae	Semalu	Leaves/Methanol	NA	62.36% at 100 μg/ml	Flavonoids, phenolic	Nguyen et al., 2004; Apaya and Chichioco-Hern, 2011
			Aerial part/Methanol	52.7	65.50% at 100 μg/ml		
*Momordica charantia*	Cucurbitaceae	Peria	Pulp/Methanol	NA	96.50% at 100 μg/ml	Flavonoid, tannin, coumarins, glycoside	Kong et al., 2000; Alsultanee et al., 2014
			Seed/Methanol		45.00% at 100 μg/ml		
*Morinda citrifolia*	Rubiaceae	Mengkudu jantan/mengkudu besar/noni	Fruit/Methanol	NA	64.00% at 0.1 mg/ml	NA	Palu et al., 2009
*Morinda elliptica*	Rubiaceae	Mengkudu hutan/mengkudu tahi ayam	Leaves/Methanol	NA	88.93% at 100 μg/ml	NA	Jamal et al., 2014
*Olea europaea*	Oleaceae	Zaitun	Leaves/Water	114,020.00	80.00% at 250 mg/ml	Oleuropein, apigenin, luteolin, caffeic acid	Al-Azzawie and Abd, 2015;Flemmig et al., 2011
			Leaves/Ethanol	42	60.00% at 50 μg/ml		
*Orthosiphon stamineus*	Lamiaceae	Misai kucing	Leaves/Ethanol	92.4	68.59% at 100 μg/ml	NA	Nguyen et al., 2004; Hendriani et al., 2016
			Aerial part/Methanol	NA	37.60% at 100 μg/ml		
*Petroselinum crispum*	Apiaceae	Daun sup	Leaves/Ethanol	NA	82.57% at 100 μg/ml	NA	Alsultanee et al., 2014; El-Rahman and Abd–Elhak, 2015
			Leaves/Methanol		28.63% at 100 μg/ml		
*Phaleria macrocarpa*	Thymelaeaceae	Mahkota dewa	Leaves/Methanol	NA	34.83% at 100 μg/ml	Phalerin	Fariza et al., 2012
*Phaseolus vulgaris*	Papilinaceae	Kacang buncis	Fruit/Water	>300	26.00% at 300 μg/ml	Flavonoids	Roohbakhsh et al., 2009
*Pimpinella anisum*	Apiaceae	Jintan manis	Fruit/Water	300.4	35.60% at 200 μg/ml	NA	Bustanji et al., 2011
*Piper betle*	Piperaceae	Sireh	Leaves/Ethanol	16.7	NA	4-allyl-1,3-hydroxychavicol	Murata et al., 2009
*Plantago major*	Plantaginaceae	Ekor anjing, daun sendok	Folium/Ethanol	NA	21.70% at 100 μg/ml	NA	Yumita et al., 2013
			Radix/Ethanol		3.66% at 100 μg/ml		
*Plumbago zeylanica*	Plumbaginaceae	Celaka putih, celaka bukit	Roots/Methanol	5	65.40% at 100 μg/ml	NA	Nile and Park, 2014
*Pogostemon cablin*	Lamiaceae	Pokok Nilam	Leaves/Methanol	NA	33.16% at 100 μg/ml	NA	Apaya and Chichioco-Hern, 2011
*Portulaca oleracea*	Portulacaceae	Gelang pasir	Leaves/Methanol	NA	39.00% at 100 μg/ml	Flavonoids, phenolic, tannins	Apaya and Chichioco-Hern, 2011
*Punica granatum*	Lythraceae	Buah delima	Seed/Methanol	NA	15.53% at 100 μg/ml	NA	Wong et al., 2014
*Salacca zalacca*	Arecaceae	Salak	Leaves/Ethanol	NA	19.66% at 100 μg/ml	NA	Azmi et al., 2012
			Pulps/Ethanol		2.88% at 100 μg/ml		
			Ripe fruit peels/ethanol		12.85% at 100 μg/ml		
*Senna alata*	Fabaceae	Gelenggang	Leaves/Methanol	NA	71.00% at 100 μg/ml	Kaempferol	Fadzureena et al., 2013
*Synsepalum dulcificum*	Sapotaceae	Buah ajaib	Fruit/Ethyl acetate	NA	80.00% at 10 mg/ml	NA	Shi et al., 2016
*Tamarindus indica*	Fabaceae	Asam jawa	Pulp/Ethanol	NA	21.40% at 100 μg/ml	NA	Yumita et al., 2013
			Lignum/Ethanol		44.90% at 100 μg/ml		
*Tetracera scandens*	Dilleniaceae	Mempelas kasar	Root and stem/methanol	33.3	73.60% at 100 μg/ml	NA	Nguyen et al., 2004
*Tinospora rumphii*	Menispermaceae	Petawali	Leaves/Methanol	NA	39.99% at 100 μg/ml	Alkaloids, terpenoids, tannins, cardiac glycosides	Apaya and Chichioco-Hern, 2011
*Trachelospermum jasminoides*	Apocynaceae	Melur hutan	Stem/Methanol	108	51.00% at 100 μg/ml	NA	Kong et al., 2000
*Vitex negundo*	Lamiaceae	Lenggundi	Leaves/Methanol	NA	50.42% at 100 μg/ml	Flavonoids, steroids, tannins, terpenoids	Apaya and Chichioco-Hern, 2011; Nile and Park, 2014
			Roots/Methanol	6	70.00%		
*Woodfordia floribunda*	Lythraceae	Seduayah	Flos/Ethanol	NA	55.33% at 100 μg/ml	Flavonoids	Yumita et al., 2013
*Zingiber officinale*	Zingiberaceae	Halia	Rhizome/Methanol	10.5 μM of 6-gingerol value	NA	NA	Alsultanee et al., 2014
			Rhizome/Water	NA	81.56% at 100 μg/ml		Nile and Park, 2014
				99,370	87.97% at 250 mg/ml		Al-Azzawie and Abd, 2015

*IC_50_ value is based on the type of solvent used in the extraction*.

*NA = data is not available*.

**Table 2:** The medicinal plants which are considered to possess anti-gout activity based on *in vivo* studies.

**Scientific name**	**Family**	**Local name**	**Part/solvent used**	**Dose of the extract**	**Experimental animal model**	**Main outcomes**	**References**
*Allium ampeloprasum*	Liliaceae	Bawang perai	Leaves/Water	5 g/kg body weight	Male albino hyperuricemia rats induced by potassium oxonate	Serum uric acid levels of hyperuricemic rats reduced significantly	El-Rahman and Abd–Elhak, 2015
*Allium cepa*	Amaryllidaceae	Bawang merah	Edible portion/Water	5 g/kg body weight	Wistar hyperuricemia rats induced by potassium oxonate	Serum uric acid levels of hyperuricemic rats reduced significantly after 14 days of treatment/onion resulted in significant inhibition on liver of xanthine oxidase activity (39.75%)	Haidari et al., 2008
*Annona muricata*	Annonaceae	Durian belanda	Leaves/Ethanol	75 mg/kg body weight	Male Wistar hyperuricemia rats induced by potassium oxonate	Serum uric acid level in oxonate-induce rats reduced significantly	Sunarni et al., 2015
				100, 200, and 400 mg/kg of body weight	Wistar hyperuricemia rats induced by potassium oxonate	All doses reduced serum uric acid levels of hyperuricemic rats by 63.98, 86.29, and 61.50%, respectively	Sri-Wahjuni et al., 2012
*Annona reticulata*	Annonaceae	Lonang, Nona kapri	Leaves/Methanol	75 mg/kg body weight	Male Wistar hyperuricemia rats induced by potassium oxonate	Serum uric acid level in oxonate-induce rats reduced significantly	Sunarni et al., 2015
*Annona squamosa*	Annonaceae	Buah nona	Leaves/Ethanol	75 mg/kg body weight orally	Male Wistar hyperuricemia rats induced by potassium oxonate	Serum uric acid level in oxonate-induce rats reduced significantly	Sunarni et al., 2015
*Apium graveolens*	Apiaceae	Saderi	Leaves/Water	5 g/kg body weight	Male albino hyperuricemia rats induced by potassium oxonate	Serum uric acid levels of hyperuricemic rats reduced significantly	El-Rahman and Abd–Elhak, 2015
			Seeds/Petroleum ether	500 mg/kg rat body weight	Male Sprague-Dawley hyperuricemia rats induced by potassium oxonate	Produced the highest reduction (56%) in uric acid level in urine	Mohamed and Al-Okbi, 2008
*Cinnamomum zeylanicum*	Lauraceae	Kayu manis	Bark/Petroleum ether	500 mg/kg rat body weight	Male Sprague-Dawley hyperuricemia rats induced by potassium oxonate	Produced the reduction (47%) in uric acid level in urine	Mohamed and Al-Okbi, 2008
*Cooccinia drandi*	Cucurbitaceae	Timun padang, pepasan	Leaves/Methanol	200 mg/kg body weight oral per day	Swiss albino hyperuricemia mice induced by potassium oxonate	Serum urate level reduced significantly up to 3.90 ± 0.07 mg/dl	Umamaheswari et al., 2007
*Dimocarpus longan*	Sapindaceae	Longan	Flower, pericarp, seed, leaf, and twig/methanol	50, 75, and 100 mg/kg of body weight	Male ICR hyperuricemia mice induced by potassium oxonate	Plasma urate levels of hyperuricemic mice reduced significantly in dose-dependent manner	Sheu et al., 2016
			Seed/Water	80 mg/kg of body weight for crude extract	Male Sprague-Dawley hyperuricemia rats induced by potassium oxonate and hypoxanthine	Serum uric acid level and xanthine oxidase activity reduced significantly. However, the extract increased xanthine oxidase activities in liver	Hou et al., 2012
*Emblica officinalis*	Euphobiaceae	Pokok melaka	Triphala powder, an Indian ayurvedic herbal formulation) (mixture of dried and powdered fruits of the three plants in equal proportions)	1 g/kg body weight oral per day	Monosodium urate crystal-induced inflammation in Swiss albino mice	Triphala treatment decreased the paw diameter significantly in monosodium urate crystal-induced mice	Sabina and Rasool, 2008
*Epiphyllum oxypetalum*	Cactaceae	Bakawali	Leaves/Ethanol and water	200, 400, 600 mg/kg body weight	Carrageenan induced adult rats of Albino Wistar strain paw edema	Percentage inhibition of rat paw edema by alcohol and aqueous extracts was 75.44 and 82.14% at dose of 600 mg/kg at 3 h	Dandekar et al., 2015
*Erythrina stricta*	Fabaceae	Bunga dedap	Leaves/Petroleum ether, chloroform, and ethyl acetate fractions	200 mg/kg body weight orally	Hyperuricemia Swiss albino mice induced by potassium oxonate	Produced significant reduction in serum urate levels and elicited significant inhibitory actions on xanthine oxidase/xanthinedehydrogenase enzyme activities in the mouse liver	Raju et al., 2012
*Hibiscus sabdariffa*	Malvaceae	Asam susur	Calyx/Water	1, 2, and 5% of *H. sabdariffa* extract	Male Sprague-Dawley hyperuricemia rats induced by oxonic acid	Extract significantly lowered uric acid by increasing uricase activity to promote uric acid excretion	Kuo et al., 2012
			Calyx/Ethanol extract, ethyl acetate fraction and water fraction	40 and 80 mg/kg body weight	Male Wistar hyperuricemia rats induced by potassium oxonate	The extract showed a significant reduction in serum uric acid leveland had uricosuric effect that increased the excretion of uric acid in urine significantly	Wahyuningsih et al., 2016a
*Jatropha curcas*	Euphorbiaceae	Pokok jarak	Roots/Methanol	100 and 200 mg/kg orally	Carrageenan induced Swiss albino mice and the Wister rat paw edema	There were dose-dependant significant reduction in carrageenan-induced rat paw edema at 100 and 200 mg/kg of extract	Mujumdar and Misar, 2004
*Leonurus sibiricus*	Lamiaceae	Pokok padang deman	Leaves/Water	50, 100, and 200 mg/kg orally	Sprague–Dawley hyperuricemia rats induced by oteracil potassium	Extract reduced serum uric acid and creatinine levels of hyperuricemia rats and promote the excretion of uric acid of kidney	Yan et al., 2014
*Mangifera indica*	Anacardiaceae	Mangga	Leaves/Ethanol	100 and 200 mg/kg body weight by oral per day for crude extract	Monosodium urate (MSU) crystals-induced gouty arthritis male Sprague-Dawley rats	Extract significantly decreased ankle swelling in monosodium urate (MSU) crystal-induced gouty arthritis rats	Jiang et al., 2012
*Orthosiphon stamineus*	Lamiaceae	Misai kucing	Leaves/Methanol	0.5, 1, and 2 g/kg body weight	Male Sprague-Dawley hyperuricemia rats induced by potassium oxonate	Extract reduced the serum urate level inhyperuricemic rats at hour 6 and showed a significant increase in urine volume and electrolytes excretion	Arafat et al., 2008
*Peperomia pellucida*	Piperaceae	Ketumpangan air/sireh cina	Whole plant with flower petroleum ether	1,000 mg/kg body weight oral per day	Carrageenan induced male Sprague Dawley rats hind paw edema	Extract showed significant in magnitude of swelling 4 h following carrageenan administration	Mutee et al., 2010
*Petroselinum crispum*	Apiaceae	Daun sup	Leaves/Water	5 g/kg body weight	Male albino hyperuricemia rats induced by potassium oxonate	Serum uric acid levels of hyperuricemic rats reduced significantly	El-Rahman and Abd–Elhak, 2015
*Phyllanthus emblica*	Phyllanthaceae	Pokok Melaka	Fruit/Alcoholic and water	200 and 400 mg/kg of body weight	Male Sprague-Dawley hyperuricemia rats induced by potassium oxonate	Both extracts showed reduction in platelets counts, serum creatinine, uric acid, blood urea nitrogen and xanthine oxidase enzyme level	Sarvaiya et al., 2015
*Phyllanthus niruri*	Phyllanthaceae	Dukung anak	Leaves/Methanol	50 mg/kg body weight oral per day	Male Sprague-Dawley hyperuricemia rats induced by potassium oxonate	Extract increased urinary uric acid excretion and exhibited a significant 76.84% inhibition of xanthine oxidase activity	Murugaiyah and Chan, 2009
*Piper nigrum*	Piperaceae	Lada hitam	Piperine (active compounds)	30 mg/kg body weight oral per day	Monosodium urate crystal-induced inflammation in Swiss albino mice	Piperine decreased the paw diameter significantly in monosodium urate crystal-induced mice	Sabina et al., 2011
*Premna serratifolia*	Lamiaceae	Buas- buas	Wood without bark/ethanol extract	300 mg/kg body weight orally per day for 14 days	Bacteria induced Wistar albino rats hind paw edema	Extract inhibited the rat paw edema by 68.32% after 21 days	Rajendran and Krishnakumar, 2010
*Synsepalum dulcificum*	Sapotaceae	Buah ajaib	Fruit/Butanol	500–1,000 mg/kg body weight per day orally	Male ICR hyperuricemia mice induced by oxonic acid potassium salt	Extract lowered serum uric acid levels and activated hepatic xanthine oxidase	Shi et al., 2016
*Zingiber officinale*	Zingiberaceae	Halia	Rhizome/Water	50 and 100 mg/kg of body weight	Hyperuricemia rats induced by potassium oxonate	Extract reduced the uric acid levels significantly in hyperuricemic rats after 14 days	Al-Azzawie and Abd, 2015
*Zingiber zerumbet*	Zingiberaceae	Halia hutan, Lempoyang	Rhizome/mixture of hexane and ethyl acetate	10 and 20 mg/kg of body weight	Carrageenan induced female Sprague dawley rats hind paw edema	10 and 20 mg/kg zerumbone exhibited significant maximum inhibition of 45.67 and 70.37%, respectively	Somchit et al., 2012

## Discussion

Medicinal plants contain many bioactive compounds and antioxidants that can be used as complementary or alternative medicines to treat gout. In fact, ~65–80% of people in developing countries use medicinal plants as remedies (World Health Organization, 2011). Plants are also important sources of medicines in the United States, where at least one plant-based ingredient is used in 25% of pharmaceutical prescriptions (Kumar and Azmi, 2014).

The xanthine oxidase inhibition assay is considered a gold standard to study the anti-gout potential of medicinal plants. Some plants and their phytochemicals are worthy of exploration as they can act as xanthine oxidase inhibitors. These compounds are also safe if an appropriate amount is taken and have few side effects (Rates, 2001; Abd Aziz et al., 2011). Previous studies have reported that five vegetables contain possible agents that can cause acute or chronic toxicities when consumed in large quantities or over a long period of time (Orech et al., 2005). Thus, it is very important for researchers to evaluate the toxicity of plants in *in vitro* and *in vivo* studies and clinical trials.

In this study, ~46 families of plants were identified and studied, both *in vitro* (*n* = 30) and *in vivo* (*n* = 24), for anti-gout activity (Tables 1, 2). Plants from the Asteraceae, Cucurbitaceae, Fabaceae, Lamiaceae, and Zingiberaceae families have been studied extensively. *Momordica charantia*, from the Cucurbitaceae, had the highest in percentage of xanthine oxidase inhibitory activity of 96.5% at 100 μg/mL using 70% methanol extract (Alsultanee et al., 2014); the total phenolic content of this plant was 80.83 ± 0.30 mg gallic acid equivalent/100 g. Further phenolic compound analysis revealed the presence of phenolic compounds, including tannin, coumarin, flavonoid, and glycoside; among these, coumarine had the strongest inhibitory activity (97.29 %) against xanthine oxidase (Alsultanee et al., 2014). Other studies have suggested that this activity is due to the presence of bioactive phenolic compounds, such as polyphenols, tocopherols, and alkaloids, in the pulp of the plant (Tan et al., 2008). However, other plants in this family, such as *Cucurbita pepo* and *Citrullus colocynthis*, have lower xanthine oxidase inhibition values of 27.33% at 100 μg/mL and 14.40% at 200 μg/mL, respectively (Bustanji et al., 2011; Alsultanee et al., 2014).

In the Zingiberaceae family, *Kaempferia galanga* had the highest xanthine oxidase inhibitory activity at 100 μg/mL (90.6%), followed by *Zingiber officinale* (81.56%), *Alpinia galanga* (57.99%), and *Curcuma longa* (28.31%) (Nguyen et al., 2004; Yumita et al., 2013; Alsultanee et al., 2014). Yumita et al. (2013) also studied *K. galanga* but the results were in contrast to other studies (28.86%). These contrary results could be due to the different localities (Vietnam and Indonesia), although both studies employed similar drying methods. Moderate total phenolic content was found in *Z. officinale*, with a value of 62.18 ± 0.65 mg gallic acid equivalent/100 g (Alsultanee et al., 2014).

Plants from the Asteraceae family include *Artemisia vulgaris, Blumea balsamifera, Chrysanthemum indicum*, and *Chrysanthemum sinense*, of which *C. indicum* exhibited 95% xanthine oxidase inhibitory activity at 100 μg/mL. The isolated flavonoid compounds from the flower of *C. indicum*, namely luteolin and apigenin, may act as xanthine oxidase inhibitors (Kong et al., 2000). Moreover, *C. sinense* also had higher xanthine oxidase inhibitory activity (82.90%) at 100 μg/mL with an IC_50_ value of 5.1 μg/mL (Nguyen et al., 2004). Further isolation of the active compounds from the flower of *C. sinense* led to the identification of caffeic acid, luteolin, eriodictyol, and 1,5-di-*O*-caffeoylquinic acid, which, among them, luteolin displayed more potent inhibitory activity compared to the positive control allopurinol, with IC_50_ values of 1.3 and 2.5 μM, respectively (Nguyen et al., 2004). *A. vulgaris* also exhibited higher xanthine oxidase inhibitory activity of 89.30% at 100 μg/mL (Nguyen et al., 2004).

Method of extraction is considered an important factor that affects xanthine oxidase inhibitory activity. The type of solvents used also contributes to differences in compounds extracted from the plants. El-Rahman and Abd–Elhak (2015) and Alsultanee et al. (2014) reported similar results on the ethanol and methanol extracts of *Petroselinum crispum*, with inhibition values of 82.57 and 28.63%, respectively. In contrast, Alsultanee et al. (2014) and Al-Azzawie and Abd (2015) reported that both the methanol and aqueous extracts of *Z. officinale* had similar xanthine oxidase inhibition percentages, with values of 81.56% and 87.97%, respectively. In addition, Azmi et al. (2012) reported that both methanol and ethanol had a higher capacity to extract xanthine oxidase inhibitors from all parts of plants; 25% of all plant extracts showed more than 50% inhibition using these two solvents compared to distilled water with only 20% of all plant extracts showing more than 50% xanthine oxidase inhibitory activity. In another study, methanol extract was found to be more active than hydroalcoholic and aqueous extracts (Nguyen et al., 2004; Umamaheswari et al., 2007). Even though methanol and ethanol extracts have higher rates of xanthine oxidase inhibitory activity, safety is the main concern of the pharmaceutical industry. Alcohol is a nervous system depressant that impairs the transmission of nerve signals, ultimately leading to respiratory suppression (Bailey and Bailey, 2000). Methanol is a highly poisonous solvent that can upset the acid-base balance of body (Azmi et al., 2012). Therefore, identifying a less toxic solvent is important.

Based on results of xanthine oxidase inhibitory activity analysis, the following plants showed more than 85% activity at 100 μg/mL: *M. charantia* (96.50%), *C. indicum* (95.00%), *Cinnamomum cassia* (93.00%), *K. galanga* (90.60%), *A. vulgaris* (89.30%), and *Morinda elliptica* (88.93%) (Kong et al., 2000; Nguyen et al., 2004; Alsultanee et al., 2014; Jamal et al., 2014). Of the other studied plants, three exhibited at least 80% activity, including *C. sinense* (82.90%), *Z. officinale* (81.56%), and *B. balsamifera* (80.90%) (Nguyen et al., 2004; Alsultanee et al., 2014; Jamal et al., 2014) at 100 μg/mL, while *Olea europaea* and *Synsepalum dulcificum* exhibited 80.00% activity at 250 mg/mL and 10 mg/mL, respectively (Al-Azzawie and Abd, 2015; Shi et al., 2016). IC_50_ values, the concentration at which half the xanthine oxidase activity is inhibited, were determined in a few studies. In this study, the lowest IC_50_ value was 0.111 μg/mL, indicating that *B. balsamifera* extract inhibited 50% of xanthine oxidase activity (Nessa et al., 2010).

A few studies further analyzed and isolated the bioactive compounds present in plants that exerted the highest xanthine oxidase inhibitory activity, allowing them to act as xanthine oxidase inhibitors by blocking the biosynthesis of uric acid from purine in the body (Unno et al., 2004). Please see the following examples: cardiac glycosides (Apaya and Chichioco-Hern, 2011), flavonoids (Nguyen et al., 2004; Roohbakhsh et al., 2009; Umamaheswari et al., 2009; Nessa et al., 2010; Apaya and Chichioco-Hern, 2011; Yumita et al., 2013), phenolics (Umamaheswari et al., 2009; Apaya and Chichioco-Hern, 2011; Sowndhararajan et al., 2012; Alsultanee et al., 2014; Al-Azzawie and Abd, 2015), anthocyanins (Al-Azzawie and Abd, 2015), tannins (Apaya and Chichioco-Hern, 2011), alkaloids (Apaya and Chichioco-Hern, 2011), proanthocyanidin A2 (Sheu et al., 2016), acetonylgeraniin A (Sheu et al., 2016), phalerin (Fariza et al., 2012), 4-allyl-1,3- hydroxychavicol (Murata et al., 2009), kaempferol (Fadzureena et al., 2013), terpenoids Apaya and Chichioco-Hern, 2011, luteolin (Kong et al., 2000; Nguyen et al., 2004; Flemmig et al., 2011), apigenin (Kong et al., 2000; Flemmig et al., 2011), caffeic acid (Nguyen et al., 2004; Flemmig et al., 2011), eriodictyol (Nguyen et al., 2004), oleuropein (Flemmig et al., 2011), luteolin-7-O–d-glucoside (Flemmig et al., 2011), and scopoletin (Ding et al., 2005). Until now, these bioactive compounds have not been further analyzed or developed into anti-gout medications.

Hyperuricemia has been modeled in pre-clinical studies by blocking uricase enzyme with potassium oxonate (Umamaheswari et al., 2007; Haidari et al., 2008). Administration of potassium oxonate (250 mg/kg) results in marked increases in serum uric acid level in rats (Shi et al., 2016). Several *in vivo* studies have demonstrated a reduction of serum uric acid levels in hyperuricemic rats. For example, administration of aqueous and alcoholic extracts of *Phyllanthus emblica* (200 and 400 mg/kg) reduced serum uric acid and xanthine oxidase enzyme levels in hyperuricemic rats while allopurinol was more potent in inhibiting xanthine oxidase enzyme (Sarvaiya et al., 2015). Similar results have also been reported by El-Rahman and Abd–Elhak (2015) for *Allium ampeloprasum, Apium graveolens*, and *P. crispum* using albino rats, where both extracts significantly reduced serum uric acid and lipid peroxidation and increased antioxidant enzyme activity levels at a dose of 5 g/kg. Phytochemical screening of the extracts also revealed their major constituents, which include phenolic (polyphenols, tocopherols, and alkaloids), flavonoids, and saponins that may act as xanthine oxidase inhibitors (Fejes et al., 2000; Zhou and Yu, 2006; Sreeramulu and Raghunath, 2010).

Some of the active compounds were isolated from the medicinal plants for investigating the underlying mechanisms of hypouricemic actions in rat model. Zeng et al. (2017) studied the bioavailability of scopoletin or 6-methoxy-7-hydroxycoumarin, a major active coumarin isolated from the stems of *Erycibe obtusifolia* and its hypouricemic effects *in vivo*. In this study, they encapsulated scopoletin into Soluplus micelles (Soluplus-based scopoletin micelles, Sco-Ms) in order to improve its oral bioavailability. To study the pharmacokinetics and biodistribution *in vivo*, the rats were orally administered with scopoletin suspension, physical mixtures of scopoletin and Soluplus (Sco-PM) and Sco-Ms at dose of 100 mg/kg scopoletin. At predetermined time intervals (2, 5, 10, 15, 20, 30, 45, 60, 90, and 120 min), the blood samples were collected for determining the plasma concentrations of scopoletin. Sco-Ms showed significantly higher maximum plasma concentration, *C*_max_ of 14,674.796 ± 2,997.147 μg/L than scopoletin and Sco-PM at 10 min. Orally administered Sco-Ms was rapidly absorbed than Sco-PM and scopoletin, with a time to reach maximum plasma concentration, *t*_max_ of 0.167 h while the time taken for plasma concentration of Sco-Ms to reduce by 50% of its initial value, *t*_*1/2*_ was 0.468 h. Sco-Ms showed CL value (ability to clear drug from the bloodstream which usually by hepatic metabolism or renal excretion) of 28.703 ± 3.482 L.h^−1^.kg^−1^. Interestingly, Sco-Ms was found to have higher scopoletin concentration in liver than the scopoletin suspension which would be importance for the inhibition of hepatic xanthine oxidase activity. The hepatic and serum xanthine oxidase activity of hyperuricemic rats were investigated in order to determine the possible mechanism of the anti-hyperuricemic effect of Sco-MS. Based on the result obtained, the oral administration of Sco-Ms at dose of 300 mg/kg reduced the serum uric acid concentration to the normal level. In addition, Zhang et al. (2016) studied the biodistribution and hypouricemic efficacy of morin (3,5,7,2′,4′-pentahydroxyflavone), a yellow pigment present in the plants from the Moraceae family. In this study, they tested a novel self-nanoemulsifying drug delivery system based on morin-phospholipid complex (MPC-SNEDDS) *in vivo* which improved the oral bioavailability of morin. After the administration of morin suspension, the concentration of morin in liver was markedly higher than other tissues (e.g., heart, spleen, lung, and kidney) at 0.5, 1, and 4 h. Moreover, the morin concentration in the liver at 0.5 h after orally administered with MPC-SNEDDS (1,096 μg/mg) was three-fold higher than morin suspension (252 μg/mg) and thus, MPC-SNEDDS possessed more potent inhibitory effect on hepatic xanthine oxidase activity than morin. As expected, MPC-SNEDDS reduced serum uric acid level of hyperuricemic rats (145 μmol/l) to normal (45 μmol/l) at 6 h after oral administration. Hence, the hypouricemic effect of the active compounds (e.g., morin and scopoletin) may therefore be explained, at least in part, by a lowering of xanthine oxidase activity in rat liver.

Another possible mechanism to reduce plasma uric acid concentration is to inhibit the reabsorption of urate in renal tissue. In some studies, the mRNA and protein expression levels of the transporters responsible for urate reabsorption are examined in order to explore the underlying molecular mechanisms of uricosuric effects of active compounds or medicinal plants. For instance, mangiferin, an isolated compound from the leaves of *Mangifera indica* significantly decreased the mRNA and protein levels of URAT1 and GLUT9 in kidney of hyperuricemic rats, suggesting that it possessed the uricosuric action, which was associated to inhibiting reabsorption of urate (Yang et al., 2015). In other study, *Dimocarpus longan* Lour seed decreased GLUT9 protein level from the liver of the rat model (Hou et al., 2012). The ethanol extract of Ramulus mori, the branch of *Morus alba* possessed the uricosuric effects in hyperuricemic mice by down-regulating renal mURAT1 and mGLUT9 expression and up-regulating renal mOAT1 expression, which contributed to the enhancement of urate excretion and reduction of serum urate level as well as improved renal dysfunction in hyperuricemic rats by up-regulating renal expression of mOCT1, mOCT2, mOCTN1, and mOCTN2 (Shi et al., 2012). In cell culture model, stably hURAT1 transfected human epithelial kidney cell line was used by Zhang et al. (2017) to evaluate the ability of tigogenin (active metabolites of dioscin) in inhibiting ^14^C-uric acid uptake via hURAT1 and the result showed that this compound possessed significant inhibitory effect from 10 to 100 μM with a concentration-dependent manner and the uric acid permeability was significantly reduced to 60% at 100 μM.

The results of standard *in vitro* screening assays provided useful information to guide the next stage of investigation such as testing the plant extract in rodents. Administration of ethyl acetate fraction from a butanol extract of *S. dulcificum* resulted in 80% of xanthine oxidase inhibitory activity at 10 mg/mL; the effects of butanol extract from this fruit was similar to the results of an *in vivo* study using allopurinol (Shi et al., 2016). Al-Azzawie and Abd (2015) showed that the *Z. officinale* extract had the highest xanthine oxidase inhibition *in vitro* (87.97%) at 250 mg/mL; at both doses (100 and 250 mg/kg), ginger extract significantly reduced mean serum uric acid levels and inhibited xanthine oxidase activity in hyperuricemia rats.

Some studies have shown that different parts of the same plants can contribute differently to effects on uric acid levels. For example, methanol extracted from the *D. longan* flowers had a greater effect on lowering uric acid compared to the seeds due to the 10 phytochemicals in the flowers. Further analysis revealed that proanthocyanidin A2 and acetonylgeraniin have higher inhibitory activity against xanthine oxidase compared to allopurinol (Sheu et al., 2016). In addition, the ethanol extract from *Hibiscus sabdariffa* calyx, as well as ethyl acetate and water fractions, reduced uric acid levels in male Sprague-Dawley rats and Wistar rats, where the ethyl acetate fraction at a dose of 6.25 mg/kg demonstrated the best effect on uricosuric compared to water fraction and ethanol. Phytochemical screening of the ethanol extract of this plant also revealed the presence of flavonoid, saponin, polyphenol, and quinone (Wahyuningsih et al., 2016b). Monosodium urate crystal-induced inflammation in mice or rats is commonly used to study the anti-gout effect of plant extracts (Sabina and Rasool, 2008). Oral administration of triphala significantly reduced paw diameter at a dose of 1 g/kg body weight (Sabina and Rasool, 2008). Extracts from the *M. indica* leaf also significantly reduced ankle swelling in monosodium urate crystal-induced gout arthritis at a dose of 200 mg/kg across 8 h (Jiang et al., 2012).

In this study, we evaluated whether the doses used in *in vitro* and *in vivo* studies are physiologically relevant. In one study, administration of 250 mg/mL of *Z. officinale* extract resulted in high levels of xanthine oxidase inhibiton (87.97%) *in vitro*, while 250 mg/kg exhibited 57.14% of xanthine oxidase inhibition and significantly reduced serum uric acid levels (Al-Azzawie and Abd, 2015). In another study, *S. dulcificum* extract administration suppressed xanthine oxidase activity in MSU-treated RAW264.7 macrophages at 500 μg/mL, while a 1000 mg/kg dose *in vivo* reduced uric acid levels in rats (Shi et al., 2016). Methanol extracts from *Phyllanthus niruri* resulted in 67.66% inhibition at 100 μg/mL in an *in vitro* study and caused significant inhibition (76.84%) of xanthine oxidase activity at a 50 mg/kg dose *in vivo* (Murugaiyah and Chan, 2009). The results from these studies were very similar results in inhibiting xanthine oxidase activity, suggesting that the doses used were physiologically relevant.

Allopurinol, common drug used for gout patients, is approved by the US FDA for doses up to 800 mg/day for the treatment of hyperuricemia and gout (Chao and Terkeltaub, 2009). One study reported that gout patients attained target serum uric acid levels of <360 mmol/L at 300 mg/day of allopurinol, and that this dose was increased up to 600 mg/day in some patients; favorable results were observed as the dose increased and it was well tolerated, such that the therapeutic goal was achieved in 92.5% of patients. These doses are therefore well tolerated in those with well-preserved renal function (RadakPerović and ZlatkovićŠvenda, 2013). However, febuxostat, a non-purine selective xanthine oxidase inhibitor, at a daily dose of 80 mg or 120 mg was reported to be more effective than allopurinol (300 mg) in lowering serum urate levels (Becker et al., 2005).

Many plants used in *in vivo* studies, including *Peperomia pellucida, Mangifera indica, Jatropha curcas, Epiphyllum oxypetalum, Zingiber zerumbet, Emblica officinalis*, and *Piper nigrum*, have exhibited anti-inflammatory activities (Mujumdar and Misar, 2004; Mutee et al., 2010; Sabina et al., 2011; Somchit et al., 2012; Dandekar et al., 2015). In addition, zerombone, which is found in the rhizome of *Zingiber zerumbet*, may act as an anti-inflammatory agent similar to non-steroidal anti-inflammatory drugs (Somchit et al., 2012). It has been proposed that phenolic compounds, such as anthocyanins and quercetin, which are found abundantly in certain plants, can inhibit xanthine oxidase activity, as they are structurally related to xanthine (Mo et al., 2007). Additional studies must be conducted on the possible mechanisms of the anti-gout activity of these medicinal plants.

In addition, there are also human clinical trials performed in gout using plant based drugs. For instance, Prasongwatana et al. (2008) investigated the effects of roselle (*H. sabdariffa)* on urinary excretions of uric acid in human models with and without renal-stone history where they found the mean levels of uric acid clearance, uric acid excretion and fractional excretion of uric acid increased significantly after consuming *H. sabdariffa* tea and then decreased to baseline level (control) at the end of the washout period in both groups, suggesting its uricosuric effect provides a long-term benefit of hyperuricemia in gouty subjects. However, the chemical constituents responsible for the anti-gout effects in this plant yet to be fully elucidated. Furthermore, the same trend of results were observed in *Orthosiphon stamineus* tea where the consumption of this tea caused an increasing of uric acid excretion (Premgamone et al., 2001). It is well understood that the increase of uric acid excretion may result in urolithiasis (development of stones in the kidney due to supersaturation of the urine with stone-forming salts). As reviewed by Butterweck and Khan (2009), they gathered the information of few plants that have been studied for the management of urolithiasis such as *H. sabdariffa, P. niruri, O. stamineus, Andrographis paniculata, Sambucus nigra, Solidago virgaurea*, and *Dolichos biflores*. For instance, Nishiura et al. (2004) demonstrated that *P. niruri* extract reduced the uric acid level as well as normalized the urinary calcium levels in calcium stone forming patients. As mentioned above, many plants had been studied for the anti-urolithiasis rather than anti-gout activities. Furthermore, there is also a very limited number of clinical studies for the anti-gout activity as compared to *in vitro* and *in vivo* studies. To the best of our knowledge, there are no human studies on the anti-gout activity specifically to xanthine oxidase inhibitor mechanism. It is further suggested that pharmacologist and clinical investigators to conduct larger randomized clinical trials of longer duration in order to determine the efficacy of plant based drugs in the treatment of gout. The doses of the plant extract, method of extract preparation, and extraction solvent must also be taken into consideration.

## Conclusion

This review summarized the potential of Malaysian medicinal plants treat gout based on research conducted over the last 17 years. Taking all results into consideration, *M. charantia, C. indicum, C. cassia, K. galanga, A. vulgaris*, and *M. elliptica* were found to have the highest xanthine oxidase inhibitory potential *in vitro*. This review suggests further research on the natural xanthine oxidase inhibitors, especially in *in vivo* studies, clinical studies, investigation of active compounds, safety of the plants as well as the pharmacokinetic and bioavailability studies, which remain to be elucidated.

## Author contributions

FA: preparing and writing the manuscript; MA: initiate the process of the review paper; AR, NA, SS, SE: check and comment the manuscript.

### Conflict of interest statement

The authors declare that the research was conducted in the absence of any commercial or financial relationships that could be construed as a potential conflict of interest. The reviewer MK and handling Editor declared their shared affiliation.
